# Significance of clinical factors as prognostic indicators for patients with peripheral T-cell non-Hodgkin lymphoma: A retrospective analysis of 252 cases

**DOI:** 10.3892/mco.2013.146

**Published:** 2013-07-12

**Authors:** WANZHUO XIE, KEYUE HU, FAN XU, DE ZHOU, WEIJIA HUANG, JINGSONG HE, JIMIN SHI, YI LUO, JIE ZHANG, MAOFANG LIN, XIUJIN YE, ZHEN CAI, HE HUANG

**Affiliations:** Department of Hematology, Bone Marrow Transplantation Center, The First Affiliated Hospital, School of Medicine, Zhejiang University, Hangzhou, Zhejiang 310003, P.R. China

**Keywords:** β2-microglobulin, bone marrow involvement, international prognostic index, lactate dehydrogenase, peripheral T-cell non-Hodgkin lymphoma

## Abstract

The aim of this study was to retrospectively analyze the significance of different clinical factors for predicting the prognosis of patients with peripheral T-cell non-Hodgkin lymphoma (PTCL) with a median follow-up of 23 months. A total of 252 PTCL patients admitted to the First Affiliated Hospital of the School of Medicine of Zhejiang University between 2005 and 2011 were retrospectively reviewed. At a median follow-up of 23 months, the overall survival (OS) rate was 23.8%. Our results revealed that the presence of B symptoms (P<0.001), Eastern Cooperative Oncology Group (ECOG) score ≥2 (P<0.001), bone marrow involvement (BMI) (P<0.001), elevated lactate dehydrogenase (LDH) levels (P<0.001), elevated β2-MG levels (P<0.001), Ann Arbor stages III/IV (P=0.007) and International Prognostic Index (IPI) ≥3 (P=0.001) were poor prognostic factors for OS and intensive chemotherapy achieved a better OS outcome compared to the CHOP treatment. In conclusion, elevated LDH and β2-MG levels, B symptoms, Ann Arbor stages III/IV, BMI, high IPIs and high ECOG scores predict an unfavorable prognosis for PTCL patients. Compared to the conventional CHOP regimen, the intensive chemotherapy treatment may improve the prognosis of PTCL patients.

## Introduction

Peripheral T-cell non-Hodgkin lymphomas (PTCLs) arising from post-thymic T cells are a heterogeneous group of neoplasms accounting for ~7–10% of non-Hodgkin lymphomas (NHLs) in Western countries, compared to 20–30% in East Asia (1,2). PTCLs usually occur in middle-aged to elderly patients and exhibit characteristics such as diffuse disease in 68%, systemic symptoms in ~45%, bone marrow involvement (BMI) in 25.8% and extranodal disease in 37% of the cases (3). The 5-year survival rate is 20–40%, which is lower compared to that of patients with corresponding aggressive B-cell lymphomas (4).

Several prognostic factors and predictive models for NHLs have been proposed (5–8) and β2-microglobulin (β2-MG) levels, lactate dehydrogenase (LDH) levels, B symptoms (fever, weight loss or night sweats), Ann Arbor stages, Eastern Cooperative Oncology Group (ECOG) performance status, bone marrow involvement (BMI), extranodal involvement (ENI) and International Prognostic Index (IPI) were extensively investigated and applied (9). However, previous studies (9) were mainly focused on diffuse large B-cell lymphomas, which is the most common type of aggressive lymphoma. Further studies are required to assess the significance of clinical prognostic factors for PTCL. Thus, previously described prognostic factors were analyzed in order to identify favorable prognostic factor combinations applicable to PTCL patients.

In the present study, we conducted a retrospective analysis of clinical characteristics from a large series of patients diagnosed with PTCL according to the criteria of the World Health Organization Classification (10) and assessed the prognostic significance of factors such as serum LDH levels, β2-MG levels, IPI and ECOG score. In addition, the overall survival (OS) rates of different treatments were compared in order to assess the clinical characteristics, treatment guidelines, survival and prognostic factors for PTCL patients.

## Patients and methods

### Patients

A total of 276 consecutive patients diagnosed with PTCL who were hospitalized in The First Affiliated Hospital of the School of Medicine of Zhejiang University between January, 2005 and December, 2011 were retrospectively reviewed. The diagnoses were confirmed by histopathological hematoxylin and eosin (H&E) staining and determination of the immunophenotype according to the World Health Organization Classification. The immunophenotype was analyzed using mouse monoclonal antibodies of variable specificities in order to detect cellular antigens in the frozen or paraffin-embedded tissue sections. The sections were stained with T-cell (CD2, CD3 and CD45) and B-cell markers (CD20 and CD79a) and disease specificity was confirmed by positive staining with one or more T-cell-specific markers, without any positive B-cell-specific marker staining. In addition, CD30 and anaplastic lymphoma kinase-1 (ALK-1) immunostaining were used for the differential diagnosis of systemic anaplastic large-cell lymphoma and CD56, CD57, TIA-1 and granzyme B were used as markers for the diagnosis of natural killer/T-cell lymphoma (NKTCL).

Complete clinical profiles were obtained from the 252 patients who completed the follow-up and the clinical data collected for each patient included age, gender, LDH and β2-MG levels, IPI, ECOG score, Ann Arbor stage, number of ENI sites, date of diagnosis and chemotherapeutic regimen. This study was approved by the Ethics Committee of the First Affiliated Hospital of the School of Medicine of Zhejiang University and informed consent was obtained from the participants.

### Clinical staging

The disease was graded according to the Ann Arbor staging system. The performance status was based on the Eastern Cooperative Oncology Group (ECOG) scale (0–4). The patient IPI scores (9) were determined and used in the survival analysis.

### Treatment

Chemotherapeutic regimens including cyclophosphamide, hydroxydaunorubicin, oncovin and prednisone (CHOP) or CHOP-like regimens (idarubicin, mitoxantrone, liposomal doxorubicin substituting for epirubicin) were employed. Intensive chemotherapies included CHOP with etoposide (ECHOP), CHOP with cytarabine (Ara-C), ifosfamide, 2-mercaptoethane sulfonate Na (Mesna), mitoxantrone and etoposide (MINE), etoposide, methylprednisolone, Ara-C and cisplatin (ESHAP), cisplatin, gemcitabine and dexamethasone (GDP), Ara-C, cisplatin and dexamethasone (DHAP) and hyperfractionated cyclophosphamide, doxorubicin, vincristine, dexamethasone and high-dose cytarabine and methotrexate (hyper-CVAD). Twenty-two patients were administered L-asparaginase in combination with the regimens mentioned above and bortezomib treatment was administered to 8 patients. Forty patients received local radiotherapy and 6 patients underwent autologous hematopoietic stem cell transplantation (HSCT).

### Statistical analysis

The OS rate was calculated from the date of diagnosis to death or the last date of follow-up. The survival curves were analyzed by the Kaplan-Meier method and the log-rank test was used for the comparison between individual clinical characteristics and survival. The factors associated with survival as determined by univariate analysis were incorporated into a Cox proportional hazards model for multivariate analysis. The analyses were two-sided and P<0.05 was considered to indicate a statistically significant difference. Data were processed with the Statistical Package for the Social Sciences software version 16 (SPSS Inc., Chicago, IL, USA).

## Results

### Patient characteristics

**Figure 1:**
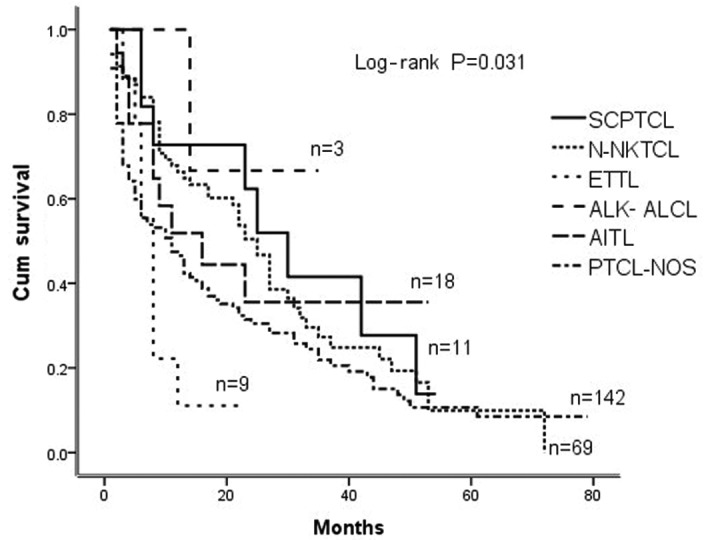
Overall survival of peripheral T-cell lymphoma (PTCL) subtypes. SCPTCL, subcutaneous panniculitis-like T-cell lymphoma; N-NKTCL, extranodal natural killer/T-cell lymphoma, nasal type; ETTL, enteropathy type T-cell lymphoma; ALK- ALCL, ALK-negative anaplastic large-cell lymphoma; AITL, angioimmunoblastic T-cell lymphoma; PTCL-NOS, peripheral T-cell lymphoma, not otherwise specified; Cum, cumulative.

The clinical characteristics of the patients are summarized in Table I. The median follow-up was 23 months (range, 1–79 months). The median age was 52 years (range, 11–77 years), with 83.3% of the patients aged <60 years. The male:female ratio was 1.90:1. A total of 156 (61.9%) patients presented with B symptoms, 95 (37.7%) had BMI and 47 (18.7%) had stage III–IV disease. The histological subtypes included 69 cases (27.4%) with nasal NKTCL, with an OS rate of 26.1%; 9 cases (3.6%) with enteropathy-type T-cell lymphoma (ETTL), with an OS rate of 11.1%; 11 cases (4.4%) with subcutaneous panniculitis-like T-cell lymphoma (SCPTCL), with an OS rate of 27.3%; 18 cases (7.1%) with angioimmunoblastic T-cell lymphoma (AITL), with an OS rate of 44.4%; 3 cases (1.2%) with ALK-negative anaplastic large-cell lymphoma, (ALK-ALCL), with an OS rate of 66.7%; and 142 cases (56.3%) with peripheral T-cell lymphoma, not otherwise specified (PTCL-NOS), with an OS rate of 24.6% (Fig. 1).

### Therapeutic results and survival analysis

**Figure 2:**
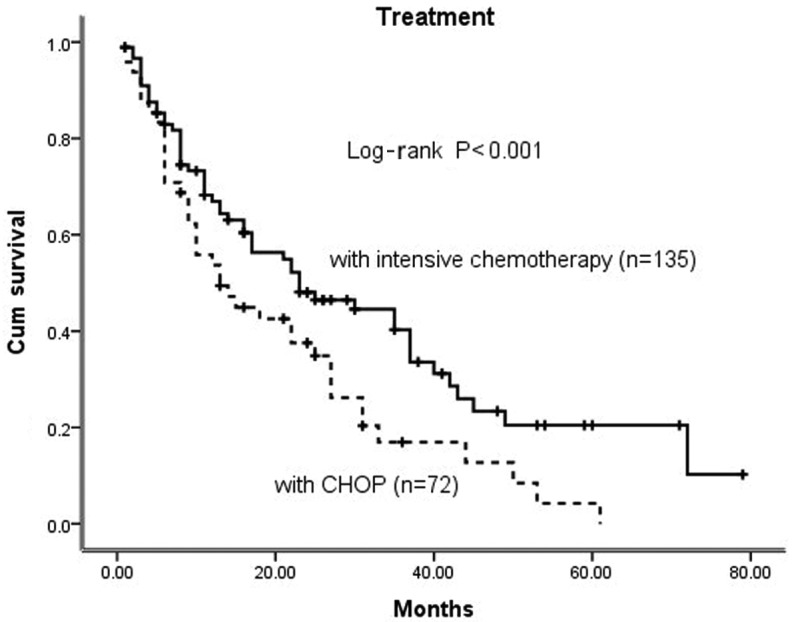
Kaplan-Meier estimates for the overall survival (OS) outcome of peripheral T-cell non-Hodgkin lymphoma (PTCL) patients with different treatments. The estimated OS rate in the intensive chemotherapy group (33.9%) was significantly higher compared to that in the CHOP therapy group (11.9%). Cum, cumulative; CHOP, cyclophosphamide, hydroxydaunorubicin, oncovin and prednisone.

Seventy-two patients (28.6%) received a combination of chemotherapy with the CHOP regimen, 135 patients (53.5%) received intensive chemotherapy and 45 patients (17.9%) refused any type of definitive therapy due to financial or personal reasons. The estimated OS rate in the intensive chemotherapy group (38.9%) was significantly higher compared to that in the CHOP group (16.7%) (Fig. 2).

### Prognostic factors

Analyses of prognostic factors are presented in Table II. A univariate analysis revealed that ECOG scores (P<0.001), B symptoms (P<0.001), Ann Arbor stages III/IV (P=0.007), BMI (P<0.001), LDH levels (P<0.001), β2-MG levels (P<0.001) and IPI (P<0.001) were poor prognostic factors for PTCL. A multivariate analysis revealed that ECOG scores (HR=3.49; 95% CI: 2.20–5.54; P<0.001), ENI (HR=0.51; 95% CI: 0.31–0.85; P=0.001), positive B symptoms (HR=1.67; 95% CI: 1.12–2.50; P=0.012), BMI (HR=0.39; 95% CI: 0.26–0.61; P<0.001) and LDH levels (HR=2.07; 95% CI: 1.40–3.06; P<0.001) were significant prognostic factors for OS.

### Association between LDH levels and prognosis

**Figure 3:**
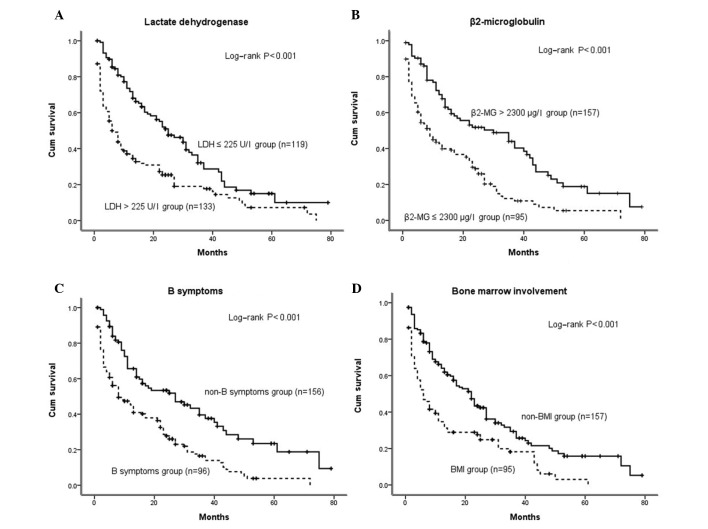
Kaplan-Meier estimates for the overall survival (OS) probability for different peripheral T-cell non-Hodgkin lymphoma (PTCL) patient groups. (A) The OS rate in the normal-level lactate dehydrogenase (LDH) group (34.5%) was significantly higher compared to that of the elevated LDH group (18.0%, P<0.001). (B) The OS rate in the normal-level β2-MG group (34.7%) was significantly higher compared to that of the elevated β2-MG group (20.5%, P<0.001). (C) The OS rate of patients with B symptoms (17.3%) was significantly lower compared to that of patients without B symptoms (39.6%, P<0.001). (D) The OS rate of patients with bone marrow involvement (BMI) (14.7%) was significantly lower compared to that of patients without BMI (32.5%, P<0.001). Cum, cumulative.

Patients were divided into normal-level (n=119) and elevated LDH (n=133)subgroups (LDH titer ≤225 and >225 U/l, respectively). The OS in the normal-level LDH group (34.5%) was significantly higher compared to that of the elevated LDH group (18.0%, P<0.001) (Table I and Fig. 3A).

### Association between serum β2-MG levels and prognosis

Patients were classified into the normal-level (n=95) and elevated β2-MG (n=157) subgroups, using 2,200 μg/l β2-MG as the cut-off point. The OS rate in the normal-level β2-MG group (34.7%) was significantly higher compared to that of the elevated β2-M group (20.5%, P<0.001) (Table I and Fig. 3B).

### Association between B symptoms and prognosis

Patients were reclassified into two groups, those with (n=96) and those without B symptoms (n=156). The OS rate of patients with B symptoms (17.3%) was significantly lower compared to that of patients without B symptoms (39.6%, P<0.001) (Table I and Fig. 3C).

### Association between BMI and prognosis

Patients were reclassified into two groups, those with (n=95) and those without BMI (n=157). The OS rate of patients with BMI (14.7%) was significantly lower compared to that of patients without BMI (32.5%, P<0.001) (Table I and Fig. 3D).

### Association between IPI and prognosis

**Figure 4:**
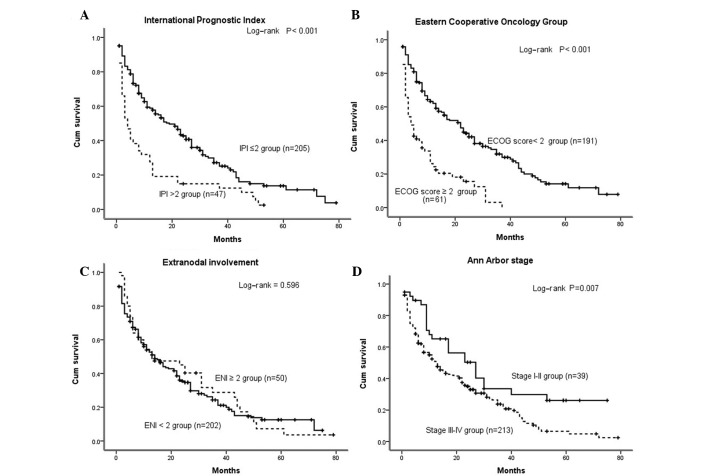
Kaplan-Meier estimates for the overall survival (OS) probability of different peripheral T-cell non-Hodgkin lymphoma (PTCL) patient groups. (A) The OS rate in the low/low-intermediate risk group (28.3%) was significantly higher compared to the intermediate-high/high risk group (19.1%, P<0.001). (B) The OS rate in the low Eastern Cooperative Oncology Group (ECOG) score (0–1) group (20.9%) was significantly higher compared to the high ECOG score (2–4) group (11.5%, P<0.001). (C) The OS rate in the group with fewer extranodal involvement (ENI) sites (27.7%) was not significantly higher compared to the group with more ENI sites (18.0%, P=0.596). (D) The OS rate in the early-stage group (I–II) (35.9%) was significantly higher compared to the late-stage group (III–IV, 24.4%, P=0.007). Cum, cumulative.

Patients were divided into two subgroups, a low/low-intermediate risk (IPI=0–1/2) and a intermediate-high/high risk group (IPI=3/4–5), comprising 184 and 68 patients, respectively. The OS rate in the low/low-intermediate risk group (28.3%) was significantly higher compared to that of the intermediate-high/high risk group (19.1%, P<0.001) (Table I and Fig. 4A).

### Association between ECOG score and prognosis

Patients were divided into two subgroups according to their ECOG scores, a 0–1 (n=191) and a 2–4 score group (n=61). The OS rate in the former group (30.9%) was significantly higher compared to that of the latter group (11.5%, P<0.001) (Table I and Fig. 4B).

### Association between ENI and prognosis

Patients were divided into 2 subgroups, an ENI<2 (n=202) and an ENI≥2 group (n=50). The OS rate in the former group (27.7%) was not significantly higher compared to that of the latter group (18.0%, P=0.596) (Table I and Fig. 4C).

### Association between Ann Arbor stage and prognosis

Patients were divided into 2 subgroups, including an early-stage (I–II) and a late-stage group (III–IV), comprising 39 and 213 patients, respectively. The OS rate in the former group (35.9%) was significantly higher compared to that of the latter group (24.4%, P=0.007) (Table I and Fig. 4D).

## Discussion

PTCLs are a heterogeneous group of neoplasms, usually diagnosed at an advanced stage and characterized by widespread dissemination, aggressive behavior and poor outcome (7). Several studies (9,11,12) were conducted in order to assess the contribution of various clinical factors to the disease prognosis. In this study, we aimed to assess the effect of age, gender, LDH and β2-MG levels, B symptoms, ENI, Ann Arbor stage, IPI and ECOG score on the prognosis of 252 PTCL patients in our hospital. It was observed that age (>60 years) was not an independent prognostic factor, which was not in accordance with the results of previous studies (8,12). A possible explanation for this inconsistency is that the median age of the enrolled patients was 52 years, which was younger compared to the cut-off age of 60 years and the age difference between the two groups was not particularly notable. In addition, the physical status of patients aged >60 years was relatively good, due to the improved overall health status of the Chinese population. Our results were consistent with those reported by previous studies (9,11,12) as regards LDH and β2-MG levels and B symptoms. These three factors were identified as good prognostic predictors and were statistically relevant with the OS rates. Therefore, it is recommended that clinicians routinely measure these factors in PTCL patients to better evaluate their prognosis and select more potent regimens for those with poor prognosis.

Among several clinical prognostic factors, the IPI seems to be suitable for peripheral T-cell lymphomas as well as diffuse large-cell lymphomas (12–14). However, in the multivariate analysis, only 3 of 5 parameters retained their prognostic significance in our study. The clinical staging and cut-off age of 60 years were no longer significant, possibly due to the disease being characterized by over four-fifths of the patients being diagnosed with advanced stage disease at presentation. The number of ENI sites was also not significant, as the location of the ENIs was more important. The presence of B symptoms and BMI have been found to correlate with prognosis and OS (14). BMI has been associated with a poor response to treatment and a shorter survival of patients with diffuse large B-cell lymphoma (15,16). In PTCL patients BMI occurs in 20–40% of the cases and appears to be associated with a worse prognosis (17). In the present study, we observed BMI in a similar proportion of cases and the resulting negative effect on survival was confirmed, whereas this effect occurred independently of other IPI prognostic factors. A previous study conducted by Gallamini *et al*(11) proposed a new prognostic model for PTCL-NOS, referred to as PIT, based on 4 simple clinical variables: age, ECOG score, LDH levels and BMI; however, further studies are required to assess the prognostic value of this model for PTCL. Although the elevated β2-MG level is not included in the IPI, there is strong evidence supporting its independent prognostic relevance in aggressive non-Hodgkin lymphomas (18). In the present study this factor was confirmed as being directly associated with poor prognosis. Although PTCL patients exhibit low survival rates, our data demonstrated that intense chemotherapy may improve their OS rates. Therefore, it is reasonable to hypothesize that intense chemotherapy may prove beneficial for PTCL patients following failure of the CHOP regimen.

In conclusion, the majority of PTCL cases present with poor prognostic characteristics at diagnosis, respond poorly to treatment and exhibit low survival rates. In particular, elevated LDH and β2-MG levels, B symptoms, Ann Arbor stages III/IV, BMI, high IPIs and high ECOG scores predict an unfavorable prognosis for PTCL patients and an intensive chemotherapy may be a comparatively more efficient PTCL treatment option.

## Figures and Tables

**Table I:** Clinical characteristics of the patients.

Characteristics	Value (%)	OS (%)	P-value
Demographics			
Age, median (years)	52		
Range	11–77		
≤60	210 (83.3)	23.5	0.765
>60	42 (16.7)	26.2	
Gender			
Male	165 (65.6)	21.8	0.142
Female	87 (34.5)	27.6	
Clinical characteristics			
ECOG score			
0–1	191 (75.8)	30.9	<0.001
2–4	61 (24.2)	11.5	
Ann Arbor stage			
I–II	39 (15.5)	35.9	0.007
III–IV	213 (84.5)	24.4	
Extranodal involvement			
<2	202 (80.2)	27.7	0.596
≥2	50 (19.8)	18.0	
B symptoms^a^			
Present	156 (61.9)	17.3	<0.001
Absent	96 (38.1)	39.6	
Bone marrow involvement			
Present	95 (37.7)	14.7	<0.001
Absent	157 (62.3)	32.5	
Serum LDH levels			
> Upper limit of normal	133 (52.8)	18.0	<0.001
≤ Upper limit of normal	119 (47.2)	34.5	
Serum β2-MG levels			
> Upper limit of normal	157 (62.3)	20.5	<0.001
≤ Upper limit of normal	95 (37.7)	34.7	
Prognostic index			
IPI risk			
Low/low-intermediate (0–1/2)	205 (81.3)	28.3	0.001
Intermediate-high/high (3/4–5)	47 (18.7)	19.1	

a B symptoms: fever, night sweats or weight loss.

OS, overall survival; ECOG, Eastern Cooperative Oncology Group; LDH: lactate dehydrogenase; β2-MG, β2-microglobulin; IPI, International Prognostic Index.

**Table II:** Risk factors for overall survival (OS).

	Univariate analysis			Multivariate analysis		
		
Variables	HR	95% CI	P-value	HR	95% CI	P-value
Age >60 years	1.07	0.67–1.72	0.771			
Male gender	0.76	0.52–1.11	0.153			
ECOG score ≥2	2.77	1.99–3.86	<0.001	3.49	2.20–5.54	<0.001
Stages III/IV	1.76	1.15–2.70	0.010			
Extranodal involvement >1	0.93	0.65–1.31	0.666	0.51	0.31–0.85	0.001
Positive B symptoms^a^	2.12	1.55–2.91	<0.001	1.67	1.12–2.50	0.012
Bone marrow involvement	0.53	0.40–0.71	<0.001	0.39	0.26–0.61	<0.001
LDH level >ULN	2.00	1.49–2.68	<0.001	2.07	1.40–3.06	<0.001
β2-MG level >ULN	2.19	1.60–3.00	<0.001			
IPI >2	2.08	1.48–2.92	<0.001			

a B symptoms: fever, night sweats or weight loss.

ECOG, Eastern Cooperative Oncology Group; LDH lactate dehydrogenase; β2-MG, β2-microglobulin; IPI, International Prognostic Index; ULN, upper limit of normal; HR, hazard ratio; CI, confidence interval.
